# A common missense variant of monocarboxylate transporter 9 (*MCT9/SLC16A9*) gene is associated with renal overload gout, but not with all gout susceptibility

**DOI:** 10.1007/s13577-013-0073-8

**Published:** 2013-08-29

**Authors:** Akiyoshi Nakayama, Hirotaka Matsuo, Takuya Shimizu, Hiraku Ogata, Yuzo Takada, Hiroshi Nakashima, Takahiro Nakamura, Seiko Shimizu, Toshinori Chiba, Masayuki Sakiyama, Chisaki Ushiyama, Tappei Takada, Katsuhisa Inoue, Sayo Kawai, Asahi Hishida, Kenji Wakai, Nobuyuki Hamajima, Kimiyoshi Ichida, Yutaka Sakurai, Yukio Kato, Toru Shimizu, Nariyoshi Shinomiya

**Affiliations:** 1 Department of Integrative Physiology and Bio-Nano Medicine, National Defense Medical College, 3-2 Namiki, Tokorozawa, Saitama 359-8513 Japan; 2 Medical Group, Headquarters, Iwo-to Air Base Group, Japan Air Self-Defense Force, Ogasawara, Japan; 3 Faculty of Pharmacy, Institute of Medical, Pharmaceutical and Health Sciences, Kanazawa University, Kanazawa, Japan; 4 Laboratory for Biofunctions, The Central Research Institute, National Defense Medical College, Tokorozawa, Japan; 5 Department of Preventive Medicine and Public Health, National Defense Medical College, Tokorozawa, Japan; 6 Laboratory for Mathematics, National Defense Medical College, Tokorozawa, Japan; 7 Department of Biology, Faculty of Science, Toho University, Funabashi, Japan; 8 Department of Pharmacy, Faculty of Medicine, The University of Tokyo Hospital, The University of Tokyo, Tokyo, Japan; 9 Department of Biopharmaceutics, School of Pharmacy, Tokyo University of Pharmacy and Life Sciences, Tokyo, Japan; 10 Department of Preventive Medicine, Nagoya University Graduate School of Medicine, Nagoya, Japan; 11 Department of Healthcare Administration, Nagoya University Graduate School of Medicine, Nagoya, Japan; 12 Department of Pathophysiology, Tokyo University of Pharmacy and Life Sciences, Tokyo, Japan; 13 Midorigaoka Hospital, Takatsuki, Japan

**Keywords:** Gouty arthritis, Single nucleotide polymorphism (SNP), Gut urate excretion, Carnitine, Solute carrier (SLC) family transporter

## Abstract

Gout is a common disease caused by hyperuricemia, which shows elevated serum uric acid (SUA) levels. From a viewpoint of urate handling in humans, gout patients can be divided into those with renal overload (ROL) gout with intestinal urate underexcretion, and those with renal underexcretion (RUE) gout. Recent genome-wide association studies (GWAS) revealed an association between SUA and a variant in human monocarboxylate transporter 9 (*MCT9/SLC16A9*) gene. Although the function of MCT9 remains unclear, urate is mostly excreted via intestine and kidney where MCT9 expression is observed. In this study, we investigated the relationship between a variant of *MCT9* and gout in 545 patients and 1,115 healthy volunteers. A missense variant of *MCT9* (K258T), rs2242206, significantly increased the risk of ROL gout (*p* = 0.012), with odds ratio (OR) of 1.28, although it revealed no significant association with all gout cases (*p* = 0.10), non-ROL gout cases (*p* = 0.83), and RUE gout cases (*p* = 0.34). In any case groups and the control group, minor allele frequencies of rs2242206 were >0.40. Therefore, rs2242206 is a common missense variant and is revealed to have an association with ROL gout, indicating that rs2242206 relates to decreased intestinal urate excretion rather than decreased renal urate excretion. Our study provides clues to better understand the pathophysiology of gout as well as the physiological roles of MCT9.

## Introduction

Gout, known for its painful arthritis, is a common disease and a consequence of hyperuricemia [1], which shows elevated serum uric acid (SUA) levels. Generally, patients can be divided into those with renal overload (ROL) gout and those with renal underexcretion (RUE) gout [2], according to their pathophysiological mechanisms of urate handling. A recent meta-analysis of genome-wide association studies (GWAS) [3] and its replication study [4] revealed that human monocarboxylate transporter 9 (*MCT9/SLC16A9*) has a relationship with SUA variation. Although the function of MCT9 is not known, it is a member of the solute-carrier (SLC) transporter that is expressed in several urate-excreting organs, including intestine and kidney. In this study, we investigated the effects of an *MCT9* variant on the susceptibility to gout in patients and healthy volunteers.

## Materials and methods

### Patients and clinical parameters for urate handling

All procedures were carried out in accordance with the standards of the institutional ethical committees involved in this project and the Declaration of Helsinki, and with written informed consent of each study participant. We collected information on 545 male gout cases from outpatients of gout clinics in Midorigaoka Hospital (Osaka, Japan). All of them were clinically diagnosed with primary gout according to criteria established by the American College of Rheumatology [5]. As a control group, information on 1,115 male individuals with normal SUA (≤7.0 mg/dl) and with no history of gout was collected from the Japan Multi-Institutional Collaborative Cohort (J-MICC) Study [6]. Mean age with standard deviation (SD) of case and control groups was 54.2 ± 13.4 and 52.6 ± 8.3 years, respectively, and mean body mass index (BMI) was 24.8 ± 3.6 and 23.2 ± 2.8 kg/m^2^, respectively. Gout patients with high (>25 mg/h/1.73 m^2^) urinary urate excretion (UUE) [2, 7–9] were defined as having ROL gout; those with low (≤25 mg/h/1.73 m^2^) UUE were described as having non-ROL gout. Cases represented as RUE gout were characterized by low (<5.5 %) fractional excretion of uric acid (FE_UA_) [2, 10] on the basis of the normal FE_UA_ range (5.5–11.1 %) [11], as previously described. Among all 545 gout cases, urate handling data, such as UUE and FE_UA_, were available in 463 cases, and the numbers of ROL gout, non-ROL gout, and RUE gout were 257, 206, and 273 patients, respectively.

### Genetic and statistical analyses

Genomic DNA was extracted from whole peripheral blood cells [12]. Genotyping of rs2242206, a common missense variant of *MCT9/SLC16A9*, gene was performed by TaqMan Assay-By-Design method (Applied Biosystems) with a LightCycler 480 (Roche Diagnostics) [13, 14]. To confirm genotypes, direct sequencing was performed with the following primers: forward, 5′-AGTGTCTGAGCTGCAAATTC-3′ and reverse 5′-CAAAAGAAATCTGCATGGAAC-3′. DNA sequencing analysis was performed with a 3130×l Genetic Analyzer (Applied Biosystems) [15]. For all calculations in the statistical analysis, SPSS v.17.0J (IBM Japan Inc., Tokyo, Japan) was used; * χ* ^2^ test was used for association analysis.

## Results

Table 1 shows result of genotyping for rs2242206 in 545 gout patients and 1,115 healthy controls. The call rate for rs2242206 was 99.9 %. Its *p* value for Hardy–Weinberg equilibrium was 0.79, which suggested mistyping was not obtained. As shown in Table 1, all minor allele frequencies (MAFs) of rs2242206 were >0.40 for all gout cases, ROL cases, non-ROL cases, and the control group, indicating this single nucleotide polymorphism (SNP) was very common in both case and control groups. Rs2242206 significantly increased the susceptibility to ROL gout cases (*p* = 0.012), with the odds ratio (OR) of 1.28, although it revealed no significant association with all gout cases (*p* = 0.10), non-ROL gout cases (*p* = 0.83), and RUE gout cases (*p* = 0.34).

**Table 1:** Association analysis of rs2242206, a common missense variant of *MCT9* gene, for gout patients

	Genotype				Allele frequency mode		
	T/T	T/G	G/G	MAF	*p* value	OR	95 % CI
Case groups							
All gout	185	247	113	0.434	0.105	1.13	0.98–1.31
ROL gout	79	117	61	0.465	0.012	1.28	1.06–1.55
Non-ROL gout	74	95	37	0.410	0.826	1.02	0.83–1.27
Control group	393	541	180	0.404	–	–	–

*CI* confidence interval, *MAF* minor allele frequency, *OR* odds ratio, *ROL* renal overload

## Discussion

This study shows that rs2242206, a common missense variant in *MCT9* gene, is associated with ROL gout but not with overall gout susceptibility. As rs2242206 (in exon 5 of *MCT9*) is close to rs12356193 (in intron 5), rs2242206 is sometimes used as a substitute for rs12356193, in which a genome-wide association with SUA was demonstrated [16, 17]. In addition, minor alleles of rs12356193 was not detected for Japanese individuals in HapMap data, and rs2242206 is a missense variant of *MCT9* (K258T), which is a possible dysfunctional mutation. We therefore investigated the relationship between *MCT9* and Japanese gout cases using rs2242206.

A previous study of a small sample (92 participants) by Polašek et al. [16] revealed no relationship between *MCT9* rs2242206 and SUA. Although our results show that rs2242206 has no association with overall gout susceptibility, rs2242206 significantly increased the risk of ROL gout. In humans, one third of urate is excreted from the intestine and most of the rest via the kidney. We previously reported that ROL is caused by decreased intestinal urate excretion due to urate transporter ABCG2 dysfunction [2]. As our study presented here shows that rs2242206 increases the risk of ROL gout but not of RUE gout, the minor allele of rs2242206 should decrease intestinal urate excretion. Our study therefore suggests that MCT9 might have a role in intestinal urate excretion: it is possible that it transports urate, but there is no report that MCT9 is a urate transporter in humans as far as we know. On the other hand, Kolz et al. [3] reported a strong triangular association among rs12356193 in *MCT9*, SUA levels, and metabolites (DL-carnitine and propionyl-l-carnitine), implying that MCT9 indirectly affects extra-renal urate excretion, for instance, by transporting carnitine-related compounds. Whereas further genetic and functional study of human MCT9 is necessary, our study suggests that it has a possible physiological role in urate excretion from human intestinal epithelial cells.
